# Feasibility Study of Soft Tooling Inserts for Injection Molding with Integrated Automated Slides

**DOI:** 10.3390/mi12070730

**Published:** 2021-06-22

**Authors:** Tobias Vieten, Dennis Stahl, Peter Schilling, Faruk Civelek, André Zimmermann

**Affiliations:** 1 Institute for Micro Integration (IFM), Faculty for Engineering Design, Production Engineering and Automotive Engineering, University of Stuttgart, Allmandring 9b, 70569 Stuttgart, Germany; zimmermann@ifm.uni-stuttgart.de; 2 Hahn-Schickard, Allmandring 9b, 70569 Stuttgart, Germany; Dennis.Stahl@Hahn-Schickard.de (D.S.); Peter.Schilling@Hahn-Schickard.de (P.S.); Faruk.Civelek@Hahn-Schickard.de (F.C.)

**Keywords:** injection molding, soft tooling, slides, prototyping, molded interconnect device

## Abstract

The production of injection-molding prototypes, e.g., molded interconnect devices (MID) prototypes, can be costly and time-consuming due to the process-specific inability to replace durable steel tooling with quicker fabricated aluminum tooling. Instead, additively manufactured soft tooling is a solution for the production of small quantities and prototypes, but producing complex parts with, e.g., undercuts, is avoided due to the necessity of additional soft tooling components. The integration of automated soft slides into soft tooling has not yet been investigated and poses a challenge for the design and endurance of the tooling. The presented study covers the design and injection-molding trial of soft tooling with integrated automated slides for the production of a complex MID prototype. The design further addresses issues like the alignment of the mold components and the sealing of the complex parting plane. The soft tooling was additively manufactured via digital light processing from a silica-filled photopolymer, and 10 proper parts were injection-molded from a laser-direct structurable glass fiber-filled PET+PBT material before the first damage on the tooling occurred. Although improvements are suggested to enhance the soft tooling durability, the designed features worked as intended and are generally transferable to other part geometries.

## 1. Introduction

Today, injection molding is one of the predominant processes for the mass production of polymer parts, ranking second only to polymer extrusion [1]. The high cost and lead times of the required hardened steel tools are redeemed over the large number of parts that can be produced with them. The situation is quite different for the production of prototypes and small series. Here, long lead times prolong the product development process by weeks and months, and tooling costs have a large share in the total costs of the individual part, so steel tools are avoided whenever possible. For ordinary injection-molding applications, developers resort to milled aluminum mold inserts, which still can produce up to tens of thousands of parts and are milled in a matter of days [2].

Molded interconnect devices (MID) are three-dimensional circuit carriers, which are typically made by injection-molding engineering thermoplastics. These thermoplastics contain a catalyst that can be activated by laser irradiation (laser direct structuring, LDS) or etching to allow a selective wet chemical metallization process in which the electrical circuit is built up [3]. Examples of the utilization of MID are the addition of electrical functions to mechanical parts to reduce the complexity of assemblies or the substitution of printed circuit boards in complex design spaces. As abrasion particles from aluminum tooling cause overplating in the wet chemical metallization process, which results in a high share of defective parts caused by short circuits, the production of MID mostly relies on steel tools [4]. As these are not suitable for the production of small series and prototypes, an alternative for milled metal tools is soft tooling. Additively manufactured soft tooling inserts have the advantage of higher design freedom (e.g., to add complex internal cooling channels) while matching production costs and lead time of their milled aluminum counterparts. A disadvantage is the shorter lifespan of soft tooling, which can vary from a few shots up to several thousand, depending on the production method and material of the mold as well as the type of material injected into them. Some of the more common additive manufacturing (AM) technologies used for the production of soft tooling are vat photopolymerization (VPP), material jetting (MJT) and material extrusion (MEX). The goals of studies using VPP technology were, e.g., the producibility of microfeatures [5,6,7], investigation of the failure mechanisms relevant for the tooling [8,9] and comparison of the performance of VPP and MJT tooling [10,11]. Other works studying MJT tooling compared it to conventional metal tooling [12,13], powder bed fusion (PBF) AM tooling [12] and resin-casting tooling [14]. Tooling made via fused filament fabrication (FFF), which is an AM process from the MEX category, was studied, e.g., for use in metal injection molding [15] and in a study describing the development of a process-specific FFF soft tooling material [16]. A good overview of the state-of-the-art concerning soft tooling in the context of tooling for injection molding is given by Dizon et al. [17]. The aforementioned PBF AM technology is typically utilized to fabricate injection-molding tooling from metal powders and thereby allows for the incorporation of highly effective complex conformal cooling channels into the tooling [18,19]. This, in turn, offers the possibility of shortened injection-molding cycle times [20]. Unfortunately, because of its powdery surfaces, PBF AM injection-molding tooling usually has to be post-processed [21], e.g., by CNC machining, to obtain the surface quality needed in the MID process, which in turn prolongs the prototyping timeframe.

**Figure 1:**
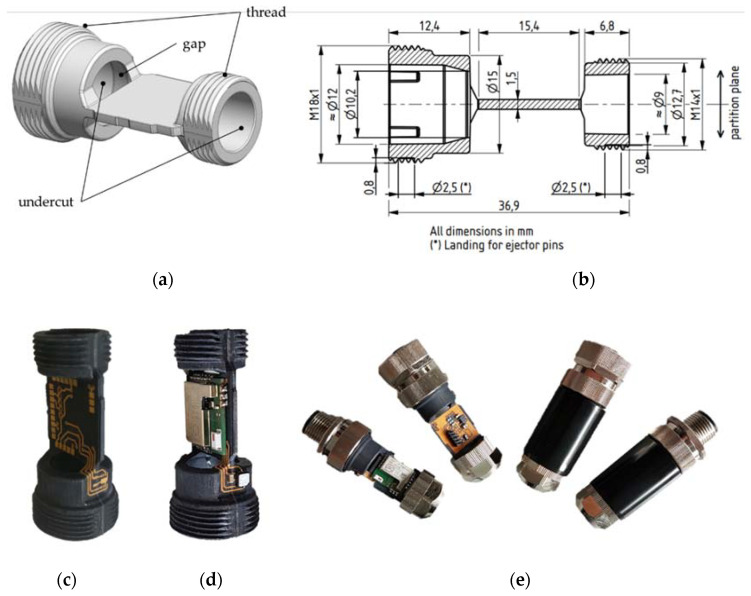
Overview on the product design: (**a**) CAD model of the molded interconnect devices (MID) base part highlighting the location of the threads, gaps and undercuts, which are features that are difficult to implement in terms of soft tooling technology; (**b**) cross-section of the part with dimensions; (**c**) bare MID showcasing the electrical layout; (**d**) MID after electronics assembly; (**e**) final product with and without a protective casing.

A lot of work is put into the reduction of tool complexity by changing the design of the part to avoid undercuts [22,23], optimizing the parting direction of the tooling [24] or employing forced demolding [25,26]. If none of these approaches is a proper solution for the desired complex part, the tooling has to be equipped with manually or automatically moved slides [25,27]. For soft tooling, the utilization of side cores, additional shaping elements that are ejected with the part and reused, has been investigated theoretically [28] as well as practically [29]. Integrating automated slides into soft tooling for the production of a complex, injection-molded part is, to our knowledge, a novel approach, the prospects of which will be investigated here. The presented study covers the design and testing of soft tooling that is used to produce prototypes of a complex MID part by employing two automatically moving slides. The MID is a component of a smart cable connector for the continuous measurement and wireless transmission of temperature and humidity sensor data [30]. The design of the MID includes two different threads of the sizes M14 × 1 and M18 × 1 and two undercuts that transition into a gap. Figure 1 shows a CAD model of the part (Figure 1a), a technical drawing of the parts cross-sectioned with the main dimensions (Figure 1b), an image of the bare MID part to showcase the electrical layout (Figure 1c), the assembled MID (Figure 1d) and the final product (Figure 1e).

A manual demolding strategy was rejected because of the laborious process, prolonged cycle times (which causes degradation of the shot in the injection unit) and the danger to damage either the part or the mold during the demolding process. Instead, the soft tooling is designed with two additively manufactured linear slides that are mechanically driven via guide pins.

## 2. Materials and Methods 

### 2.1. Design

**Figure 2:**
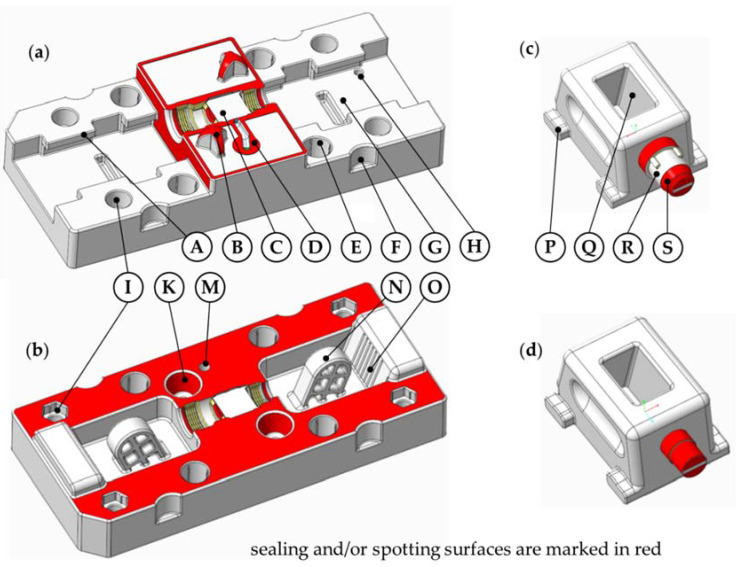
Overview of the soft tooling components and features (CAD): (**a**) ejector mold; (**b**) injection mold; (**c**) slide M18 side; (**d**) slide M14 side. (A) Guiding for a slide. (B) Mold alignment dome. (C) Cavity. (D) Sprue puller hole, runner and gate. (E) Seat for overload protection stud. (F) Seat for fastening bolt (floating). (G) Slide run. (H) Seat for spring-loaded thrust pad. (I) Seat for fastening bolt (end position). (K) Mold alignment depression. (M) Sprue hole. (N) Guide pin. (O) Slide load absorber with crush ribs. (P) Guide element. (Q) Slide guide hole(R) Part of the cavity. (S) Sealing surface for recess.

The presented soft tooling consists of four components, namely, the injection mold, the ejector mold and two different slides. The two mold halves are designed to be inserted in a modified steel mold base. The four components are equipped with standard parts like nuts, studs and spring-loaded thrust pads to obtain a functional tooling system. The overall dimensions of the assembled tooling are 179.5 mm × 80 mm × 56 mm, whereas the part itself is only about 37 mm long and has a maximum diameter of 18 mm. Shot material is fed by the injection unit through the sprue hole from the back of the injection mold, and is then redirected 90° by the ejector mold geometry into the parting plane where it moves along the 20 mm long runner and finally enters the cavity through the 0.92 mm^2^ large gate. The ejector mold features 3 ejector holes, one in each thread (Ø1.9 mm) and one for the sprue puller (Ø2.9 mm). An overview of the tooling design and its features is given in Figure 2.

**Figure 3:**
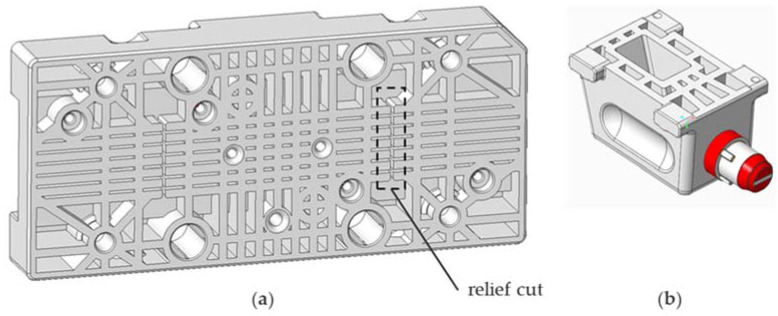
View on selected components’ backsides and the incorporated design features aimed at reducing warpage, i.e., lightweight shell design with ribbing and relief cuts: (**a**) ejector mold; (**b**) slide M18 side.

Digital light processing (DLP) is based upon the regional polymerization of liquid resin, which shrinks during this process. Therefore, parts with large material accumulations are prone to warping [31]. To minimize geometrical errors, the design follows lightweight construction design rules and long continuous stretches are divided into sections by relief cuts. Figure 3 shows the backside of the ejector mold and the M18 side slide to showcase the lightweight design of the components. In addition, the guiding structures for the slides are tolerated rather generously to avoid seizing (0.25–0.3 mm air gap).

As the dimensional accuracy of the DLP AM process in combination with the utilized resin is not good enough to mount the inserts unaltered on the mold base, they usually either are milled to a precise shape first or have to be manually positioned by shimming them with feeler gauge strips. Both are iterative processes in which the alignment is checked in between. Instead, the presented design incorporates self-aligning features to ensure the correct movement of the slides and the correct geometry of the injection-molded part. The features are designed as a trinomial dome and a corresponding pit (see Figure 2 at marking B and K). Although DLP only has average dimensional accuracy due to shrinkage and overexposure (typically in the range of 0.1 mm–0.5 mm depending on the nominal value), self-aligning features in the mold halves are possible because it has a good positional tolerance for structures that are adjacent.

**Figure 4:**
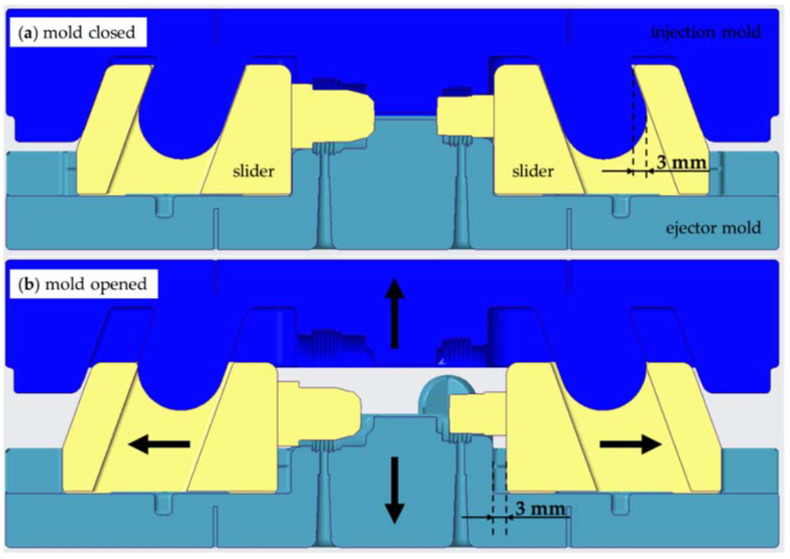
Schematic illustration of the automated slide movement: (**a**) when the mold is closed, the guide pin and the slide overlap by 3 mm; (**b**) after opening the mold, a corresponding gap of 3 mm formed between the slide and the ejector mold.

To produce the undercut geometry of the part, the tooling utilizes linear slides, which are moved both by the machine via guide pins (high forces) and manually by hand (low forces). Upon closing of the mold base, the inserts are first moved 5 mm by hand. The injection-molding machine then drives them another 3 mm via the guide pins to close the cavity completely and hold it shut against the injection pressure. When the mold base opens, the slides are again driven 3 mm by the machine via the pins and then another 5 mm by hand to clear the molded part. Figure 4 shows a schematic of the automated movement of the slides for the transition from the closed (Figure 4a) to the opened (Figure 4b) tool. The application of manual movement is the result of a compromise between the stability and the length of the guide pins. As there was no firsthand experience with soft tooling guide elements and their mechanical strength, the guide elements were designed short to prevent excessive side loads, which in turn limits the maximum automated travel distance of the slides in the parting plane. The slides are derived from a standard mechanical slide system, which allows for linear movement of the slides inside the parting plane [1]. Instead of the typical round shape, the guide pins are designed with a lightweight rectangular cross-section. To ensure correct closing of the slides, the opposing mold halve puts pressure on them via crush ribs right before the whole tool closes (0.1 mm pretension).

The milling and grinding processes applied in the production of steel molds lead to very even and precise surfaces and allow for a material-tight cavity. The surfaces of the additively manufactured soft tooling are not as even, and thus shot material leaking into the parting plane is an issue that has to be addressed. Here, the sealing of the complex parting plane was realized by compressing the mold, a method favored by the comparatively lower Young’s modulus of the polymer inserts compared to steel. Inserted steel studs protect the soft tooling inserts from the high closing forces of the injection-molding machine. The studs are ground to a specific length so they can absorb the closing forces after the inserts have been compressed by a certain amount. The effect is further enhanced by removing 0.3 mm of material in the parting plane of the ejector mold with the exception of a narrow strip around the cavity, thereby producing a sealing ring (see Figure 2a).

### 2.2. Tooling Production and Assembly

The soft tooling components were produced from the material PLASTCure Rigid 10500 (Prodways Materials, Friedberg, Germany) on a ProMaker L5000 DLP unit (Prodways, Les Mureaux, France). The material consists of a mixture of epoxy and acrylate resins filled with spherical silica particles. It was chosen for its high Young’s modulus and good thermomechanical properties (e.g., comparably low coefficient of thermal expansion). After cleaning and post-curing the components, the remainder of the DLP support structures was carefully removed with a file. Further, the ejector holes were reamed to obtain their final diameter: 2.5 mm for the ejector pins embedded in the thread geometry and 3.5 mm for the sprue puller. The diameters are equal to those of the ejector pins to ensure a leak-tight fit, which cannot be guaranteed by the tolerances of the additive manufacturing process alone. Additionally, threads were cut by hand into the four seats of the spring-loaded thrust pieces.

Before assembling the tooling, the actual width, length and height of the components were measured with a digital caliper and compared to the nominal values. The amount of warping was examined by determining the evenness of the mold halves. This was done by measuring nine evenly distributed points on the backside with a dial gauge. A drawing of the characteristical dimensions along with the results of the measurements is provided as a Supplementary Materials.

The assembly process of the soft tooling was executed as follows: nuts and overload protection studs were inserted into their respective seats, which are equipped with crush ribs. The spring-loaded thrust pads were screwed in the pre-tapped holes. Each mold half was secured in the mold base with four bolts which allowed limited lateral movement. Four additional bolts then locked the position of the ejector mold. By closing the mold base, the two mold halves self-aligned via a dome and a corresponding depression that are implemented into the soft tooling. At this stage, the injection mold was still loosely held by friction and could be locked into place with additional bolts from the back of the mold base. The two slides were mounted in their respective seats in the injection mold and pushed towards the cavity until the spring-loaded thrust pads locked them in place. All surfaces belonging to the slide system as well as the ejector pins were greased to reduce friction and prevent seizing.

### 2.3. Injection-Molding Trial

The injection-molding trials were conducted on the injection-molding machine Arburg Allrounder 270A (Arburg GmbH + Co KG, Loßburg, Germany), which uses a center feed. It is opened and closed with an electrically driven toggle joint, has a tie bar clearance of 270 mm × 270 mm and a closing force of 350 kN. The shot material is Pocan DP T 7140 LDS (Lanxess Deutschland GmbH, Cologne, Germany), a PET+PBT blend with 44% glass fiber/mineral content and an additive for the LDS MID process [32]. The process parameters that were used in the trial are given in Table 1. Before the trial started, the tooling was heated to 60 °C via a conventional fluid channel in the mold base connected to a heated water circuit. During the trial, the temperature control in the mold base continued, but as the polymer material of the soft tooling is a relatively poor conductor for heat, the dominant mechanism to remove heat from the tooling was by convection while the mold was open between shots.

The goal was to obtain 10 prototyping parts in an initial run. To test the possibility of using the mold on different production days, the machine was then shut down, and the tooling cooled to ambient temperature. After heating everything to operating temperatures once again, the trial commenced until the destruction of the soft tooling.

## 3. Results

### 3.1. Tooling Production and Assembly

**Figure 5:**
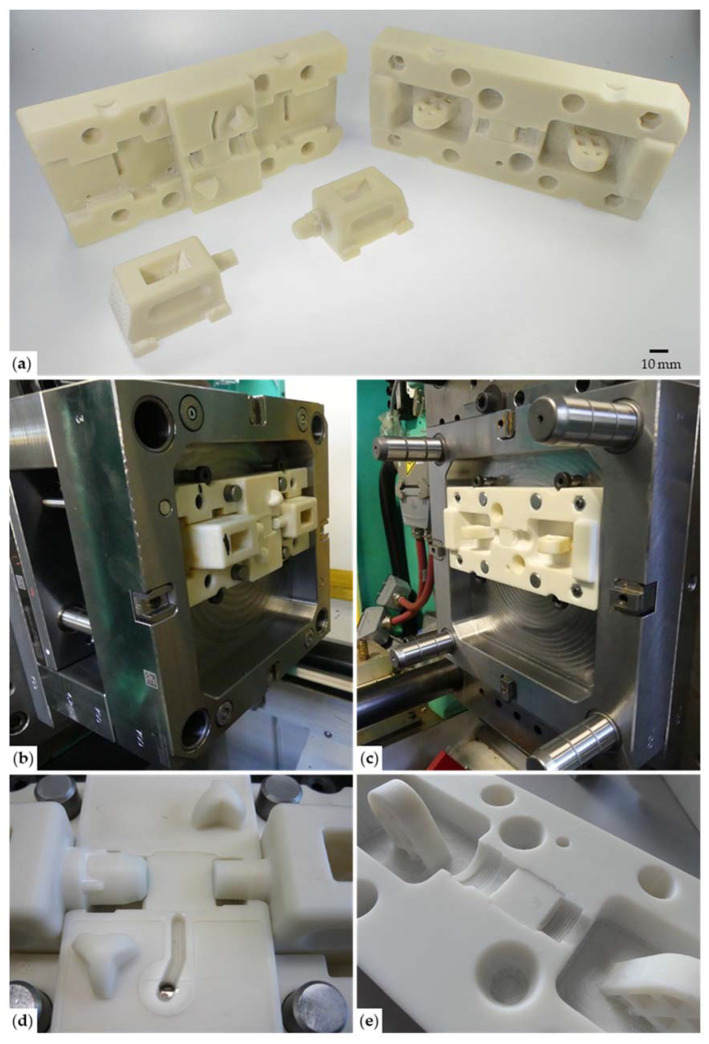
Injection-molding test setup: (**a**) overview on the soft tooling components ejector mold (**left**), injection mold (**right**) and the two slides (**front**); (**b**) ejector mold and the two slides assembled, mounted in the mold base and installed on the injection-molding machine; (**c**) injection mold mounted in the mold base and installed on the injection-molding machine; (**d**) close-up of the cavity formed by the ejector mold and the two assembled slides; (**e**) close up of the injection-mold cavity.

The tooling components were manufactured and assembled as described in Section 2. After additive manufacturing and post-processing, which took about 8.5 h and 3 h, respectively, the manual preparation and assembly steps on the soft tooling were completed in about 8 h. As the additive manufacturing process runs mostly without operator supervision and all components can be manufactured simultaneously, the fabrication of the tooling can be realized in less than 2 working days. We estimate, based on former projects executed in steel tooling, that the time for CNC machining, adjustment and assembly of steel tooling of similar complexity would have been at least around 10–15 working days. Figure 5 shows photos of the soft tooling on and off the injection-molding machine. Figure 5a highlights all the soft tooling components before the assembly, whereas in Figure 5b,c, the components are mounted in the injection-molding machine ready for use. Figure 5d,e show close-ups of the cavity from the ejection mold (with the slides mounted) and the injection mold, respectively. 

Table 2 contains the summary of the length measurements in the form of maximum dimensional deviations. The deviations are the result of overexposure by the DLP unit and shrinkage of the material upon polymerization. The variation in length and width is very similar for both tools, which is based on the geometrical similarities of the two mold halves. The evenness was determined to be 0.35 mm for the injection mold and 0.46 mm for the ejector mold. For a more instructive recapitulation of the measurements, please refer to the Supplementary Materials accompanying this study.

### 3.2. Injection-Molding Trial

**Figure 6:**
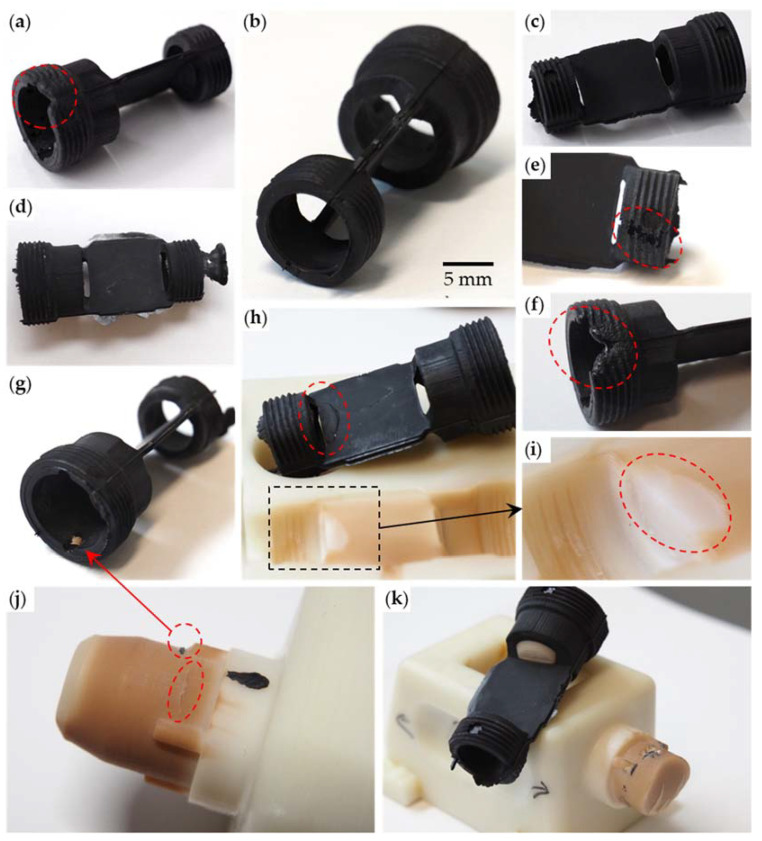
Overview on the produced parts: (**a**) first produced part with defect due to incomplete filling; (**b**,**c**) defect-free part; (**d**) part with flash; (**e**,**f**) part 18 with defects due to prolonged dwell time of the material in the injection unit; (**g**) chipped-off particle of the tooling stuck in part 17; (**h**) chipped tool and corresponding part 12; (**i**) enlarged view of the chipped tool; (**j**) slide (M18 side) with defect and crack corresponding to part 17; (**k**) tip of slide (M18 side) stuck in part 26 and the corresponding broken slide.

The injection-molding machine was heated up, and the reference point of the machine was set, first without the slides present, then with mounted slides. To enter a proper cycle sequence (i.e., thermal management of the shot and the tooling) without damaging any component, the injection-molding trial was started by injecting five shots of material before mounting the slides in the tooling. The greased slides were then mounted in the tooling, and the main trial was started. The first part was not fully formed and had defects in the M18 thread, see Figure 6a. This might have been caused by deterioration of the material in the injection unit due to a prolonged cycle time during the first assembly of the slides. The next 10 parts (parts 2 to 11) were successfully molded, see Figure 6b,c, which show correctly formed parts except for the occasional flash (Figure 6d). The flash, however, could be removed easily. After molding part 11, the machine was shut down, and the trial was continued after cooling down the whole machine and warming it up again. The slides were mounted in the tooling, and the machine was closed to reset its reference point. After opening the machine, a defect was visible in the soft tooling. A piece of the injection mold chipped off where the tip of the M14 side slide presses on the mold halves to seal and form the recess, see Figure 6h,i. The defect is also visible in the parts that were molded afterward, starting from part 12. During the production of part 17, another piece chipped off the slide on the M18 side of the cavity. A closer inspection of the slide tip also revealed a crack in the material. Figure 6g,j show the chipped mold piece stuck in part 17 and the cracked slide, respectively. A prolonged cycle time, during which shot material in the injection unit deteriorated, caused the production of a faulty part before injection of part 18, see Figure 6e,f. The final failure of the tooling occurred when the tip of the M18 side slide became stuck in part 26 during production and broke off; see Figure 6k. 

## 4. Discussion

The determined evenness of 0.46 mm on the ejector mold and 0.35 mm on the injection mold were manageable in the context of the tooling system. Due to the lower rigidity of the soft tooling material, the force of the bolts is sufficient to push them flat against the mold base and prevent damage by cyclic flexing during the injection-molding process. Although the measurements in the case of the presented tooling show very similar deviations in length and width, this would probably not be the case if the mold halves had different sizes. The maximum deviation of almost 0.5 mm indicates that without a milling process or self-aligning features, the soft tooling cavity could have a significant misalignment in the parting plane, which in turn might lead to defective parts. Since the produced parts do not show any visible offset in the region of the parting plane, the incorporated self-aligning features performed as expected.

As was planned, 10 proper parts could be produced in the initial test run. The quality of the parts was assessed by three qualitative criteria. First, the geometry should not have any defects caused by the molding process, e.g., misplaced joint lines, damage from the ejector pins or unfilled geometry. Second, the dimensions need to be accurate enough to allow for the assembly of the M14 and M18 cable endings as well as the housing. Third, the surface should not deviate more than ±300 µm from the designed geometry, otherwise, the laser beam would not ablate the material correctly during the laser-direct structuring process (the layout is based on the CAD geometry and is not corrected for deviations in the actual part geometry). This, in turn, would result in defective circuits on the MID. After a visual inspection and the functional testing of the finished products, it is evident that all three criteria were met by the 10 produced parts. The first damage to the soft tooling occurred while resetting the reference point of the machine after cooling it to ambient temperature and reheating it according to the process parameters. It is probable that, due to the temperature fluctuation, the shrinking and expanding of the materials either caused internal stresses which promoted the damage, or it resulted in a misalignment of the components, which in turn lead to the described damage when the machine was closed and the slides were pushed into the sealing surfaces. In any case, the result shows that multi-component soft tooling should be operated as continuously as possible to prevent damage from such influences. Although the maximum number of producible parts in a continuous operation is yet unknown, the presented amount is deemed sufficient for prototyping purposes or the production of customized products. Furthermore, the endurance of the soft tooling components depends heavily on the part geometry and cannot be transferred to different applications directly.

Critical failure of the soft tooling occurred during the production of part 26 when the tip of the M18 side slide broke off during demolding after the part shrank around it. This is an inherent weak point of the design because melt flows around almost the whole surface of the tip, and heat builds up inside it due to its low mass and small cross-section. This, in turn, weakens the polymer material over time. The endurance of the soft tooling could be improved by removing heat via conformal cooling channels [33] or a material that has improved heat-conducting properties [34]. The motion of the slide prohibits the incorporation of a fluidic cooling system. If changing the formulation of the AM resin is not an option, a possible alternative could be to e.g., insert a solid copper core during additive manufacturing which adds mechanical strength and also improves the heat transfer away from the tip, and therefore also improves the endurance of the tooling system overall.

The guide elements for the slides performed as intended and did not show any signs of wear or damage. Based on this experience, future soft slides can be designed more aggressively and, depending on the geometry of the part, automated slides without the need for manual intervention of the machine operator seem feasible.

During prolonged cycle times, the utilized PET+PBT shot material is prone to heat-induced deterioration in the injection unit, which leads to the production of defective parts. A non-negligible part of the cycle time was spent on the manual slide movement as described in Section 2, which left little room for errors. Since no damage or wear was detected on the guiding system of the slides, the pins could possibly be lengthened to allow for fully automated slides, which in turn would shorten the cycle time, reduce manual labor and eliminate a source of errors.

Even though the DLP material PLASTCure Rigid 10,500 shows mostly plastic behavior when it breaks, elastic compressing of the sealing surfaces did work. Some flash formed on the parts, but was easily removable by hand. For this, during the commissioning of the soft tooling, the origin of the injection-molding machine had to be adjusted quite often in order to fine-tune the length of the metal studs that protect the tooling from the closing forces. In the future, the maximum closing force permitted to compress the sealing surfaces might be calculated via the acceptable compression load of the soft tooling material. Then, the metal studs could be omitted, which would reduce tool complexity and commissioning time.

## 5. Conclusions

The production of complex polymer parts with, e.g., undercuts via injection molding using soft tooling inserts, poses a challenge for the design and endurance of the tooling components. This study explores the design and testing of a set of soft tooling components incorporating automated slides for the production of a complex molded interconnect device with undercuts, recesses and threads. Besides the slide system, the design also employs self-aligning features to address a simplified assembly of the components in the mold base, a sealing strategy for the complex parting plane and geometries for the reduction of warping induced from shrinkage during the fabrication of the components via digital-light-processing additive manufacturing. In general, the presented features worked as intended and are not product-specific, so they can be modified to be incorporated into other soft tooling projects. The soft tooling was fabricated from a silica-filled photopolymer, and, for prototyping purposes, 10 proper parts were produced during injection-molding trials from a PET+PBT material with 44% glass fiber/mineral content and an LDS additive. Although 10 parts can be sufficient for prototyping purposes or the production of individualized items, the endurance of the soft tooling components should be improved. First, damage of the mold occurred during resetting the machine after cooling the tool components to ambient temperature and reheating them, which leads to the conclusion that multi-component soft tooling should be operated as continuously as possible to obtain a higher yield.

For future studies, a cooling mechanism could be integrated into the slides and main inserts to enhance the endurance of the soft tooling. Additionally, the sealing strategy and slide system performed well, so future designs could be dimensioned more aggressively to enable an operation without manual intervention. Finally, a simulation of the tooling and the injection process might permit the omission of the protective metal studs and thus reduce the amount of commissioning work.

## Figures and Tables

**Table 1:** Process parameters for the injection-molding trials with Pocan DP T 7140 LDS material on an Arburg Allrounder 270A unit.

Parameter	Value
Injection temperature	320 °C
Tool temperature	60 °C
Injection rate	15 cm^3^/s
Injection pressure	200 MPa
Changeover point	2.4 cm^3^
Holding rate	27 cm^3^/s
Holding pressure	2.5 MPa
Holding time	2.7 s
Cooling time	14 s
Cycle time	50 s

**Table 2:** Maximum deviation from the main dimensions (length, width, height) in mm, measured with a digital caliper. For additional information on the measurements, please refer to the Supplementary Materials accompanying this study.

Component	Maximum Deviation in mm *		
	Length	Width	Height
Ejector Mold	−0.40 (179.5)	−0.07 (80)	0.22 (31)
Injection Mold	−0.46 (179.5)	−0.07 (80)	0.16 (25)
Slide M18 Side	0.20 (50)	−0.17 (38)	0.01 (30.5)
Slide M14 Side	0.02 (50)	−0.18 (38)	0.01 (30.5)

* Brackets show nominal dimensions.

## Supplementary Materials

The following are available online at https://www.mdpi.com/article/10.3390/mi12070730/s1, PDF1: Measurements Soft Tooling Components. The STL files of the soft tooling components can be requested from the corresponding author.
