# Supplementary material for: Feasibility Study of Soft Tooling Inserts for Injection Molding with Integrated Automated Slides
Source: Micromachines (Basel). 2021 Jun 22;12(7):730. doi: 10.3390/mi12070730 (PMC8306434; doi:10.3390/mi12070730)
Supplement: Supplementary file 1 [file micromachines-12-00730-s001.zip › micromachines-1254441-supplementary.pdf]

# Ejector mold

Measured with digital caliper:

| nominal | measurement | | |
|---|---|---|---|
| | **left** | **center** | **right** |
| 23.00/31.00 | 23.12 | 31.22 | 23.13 |
| deviation | + 0.52 % | + 0.71 % | + 0.57 % |
| 80.00 | 80.04 | 79.93 | 79.99 |
| deviation | + 0,05 % | - 0,09 % | - 0,01 % |
| | **top** | **center** | **bottom** |
| 179.50 | 179.10 | 179.10 | 179.16 |
| deviation | - 0.22 % | - 0.22 % | - 0.19 % |

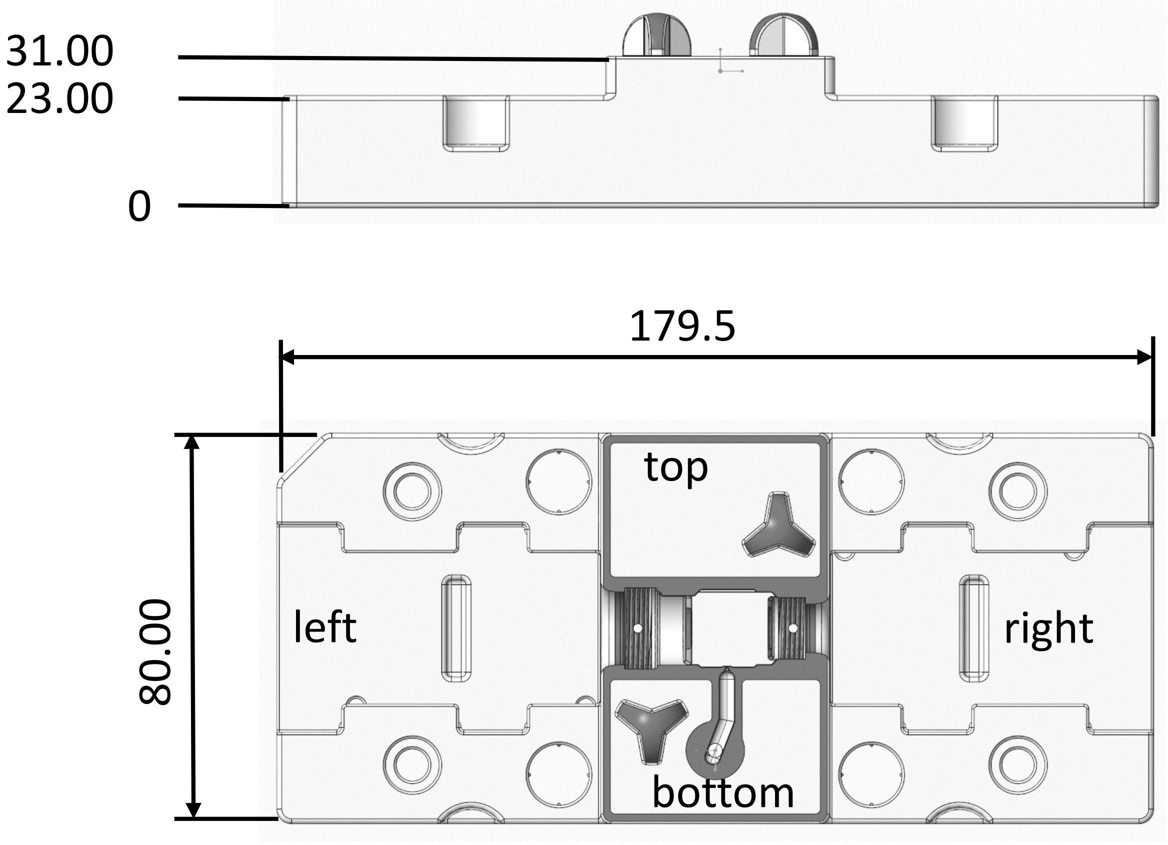


Tobias Vieten, Dennis Stahl, Peter Schilling, Faruk Civelek and André Zimmermann, Feasibility study of soft tooling inserts for injection molding with integrated automated slides, micromachines, 2021

# Injection mold

Measured with digital caliper:

| nominal | measurement | | |
|---|---|---|---|
| | **left** | **center** | **right** |
| 25.00 | 25.10 | 25.16 | 25.09 |
| deviation | + 0,40 % | + 0,64 % | + 0,36 % |
| 80.00 | 79.93 | 79.93 | 79.95 |
| deviation | - 0,09 % | - 0,09 % | - 0,06 % |
| | **top** | **center** | **bottom** |
| 179.50 | 179.04 | 179.04 | 179.05 |
| deviation | - 0,26 % | - 0,26 % | - 0,25 % |

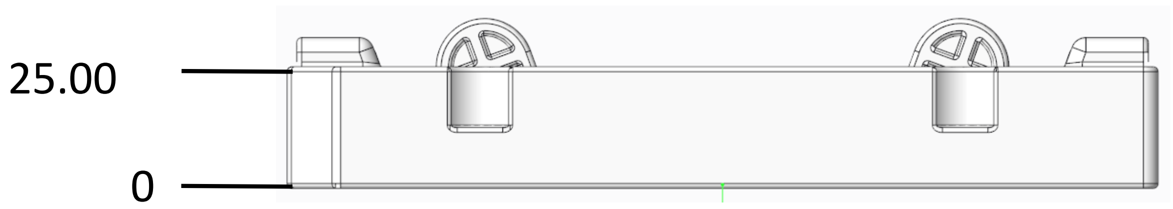


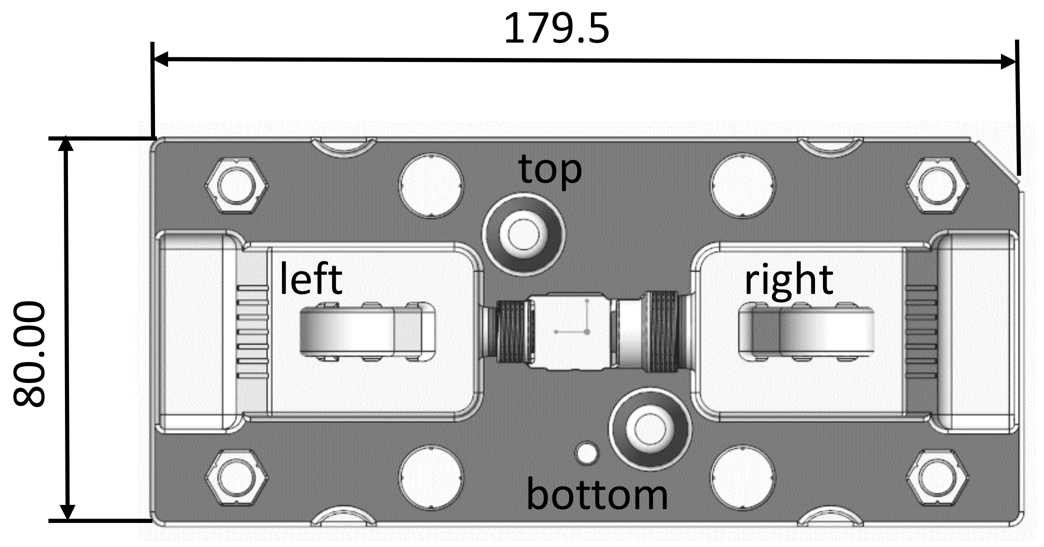


Tobias Vieten, Dennis Stahl, Peter Schilling, Faruk Civelek and André Zimmermann, Feasibility study of soft tooling inserts for injection molding with integrated automated slides, micromachines, 2021

# Slider M18 side

Measured with digital caliper:

| nominal | measurement | |
|---|---|---|
| 30.50 | 30.51 | |
| deviation | + 0.03 % | |
| 50.00 | 50.20 | |
| deviation | + 0.40 % | |
| | **front** | **back** |
| 38.00 | 37.83 | 37.92 |
| deviation | - 0.45 % | - 0.21 % |

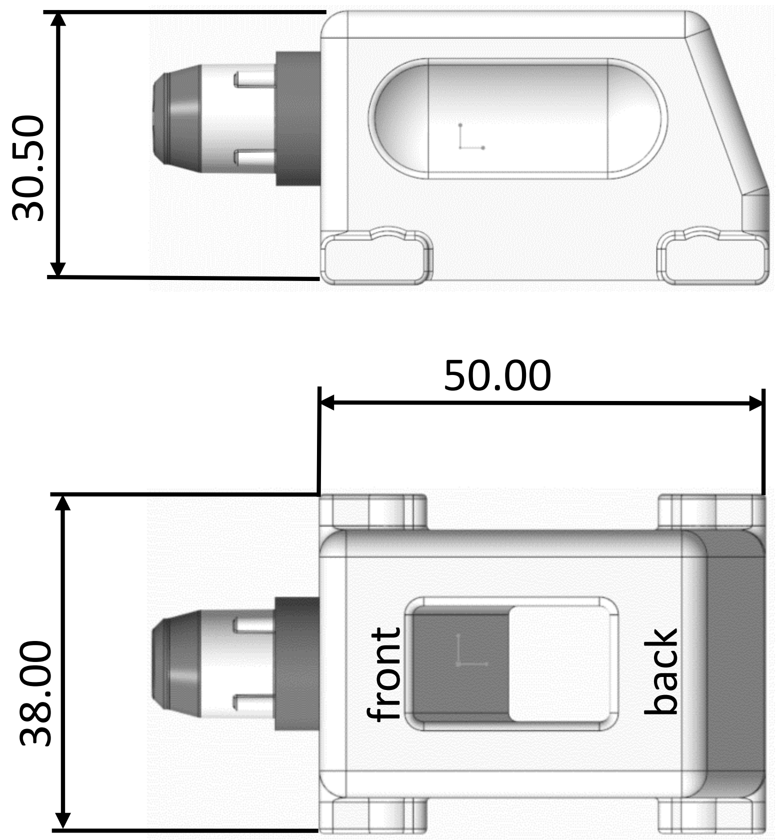


Tobias Vieten, Dennis Stahl, Peter Schilling, Faruk Civelek and André Zimmermann, Feasibility study of soft tooling inserts for injection molding with integrated automated slides, micromachines, 2021

# Slider M14 side

Measured with digital caliper:

| nominal | measurement | |
|---|---|---|
| 30.50 | 30.51 | |
| deviation | + 0.03 % | |
| 50.00 | 50.02 | |
| deviation | + 0.04 % | |
| | **front** | **back** |
| 38.00 | 37.82 | 37.89 |
| deviation | - 0.47 % | - 0.28 % |

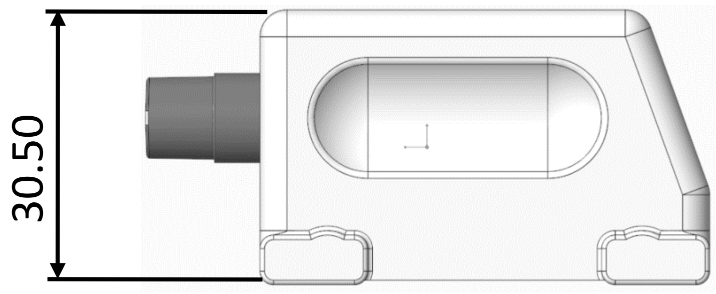


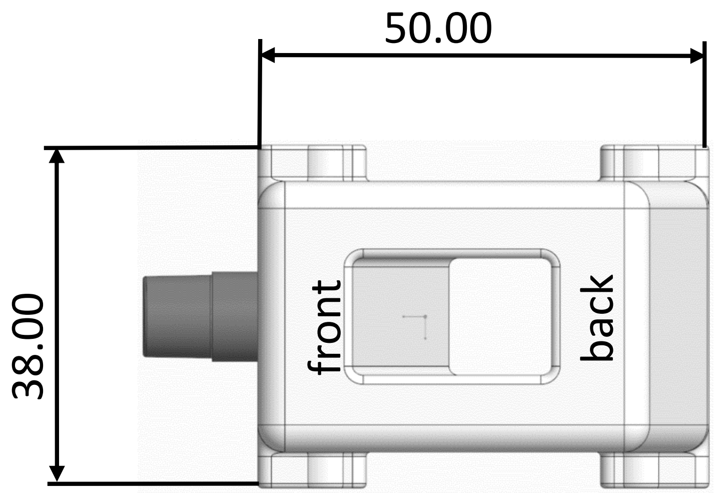

# ejector mold

Measured with dial gauge:

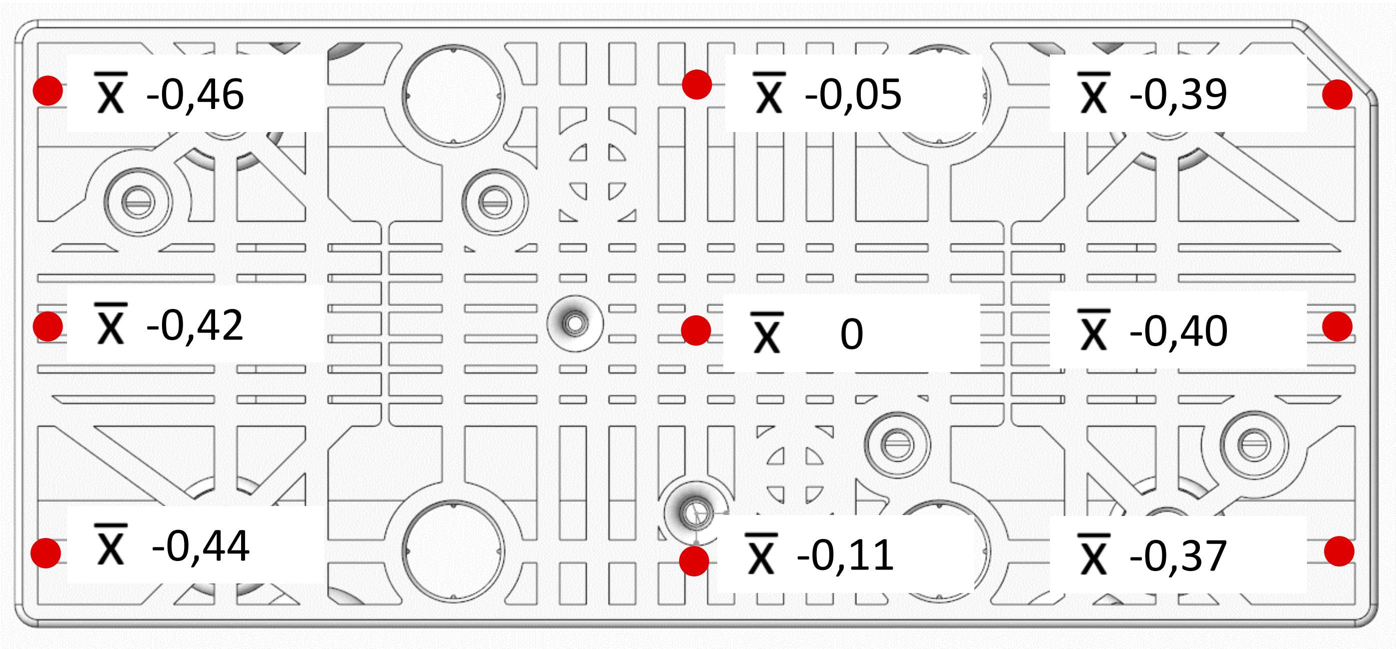


determined evenness ≈ **0,46**

**Tobias Vieten, Dennis Stahl, Peter Schilling, Faruk Civelek and André Zimmermann, Feasibility study of soft tooling inserts for injection molding with integrated automated slides, micromachines, 2021**

# injection mold

Measured with dial gauge:

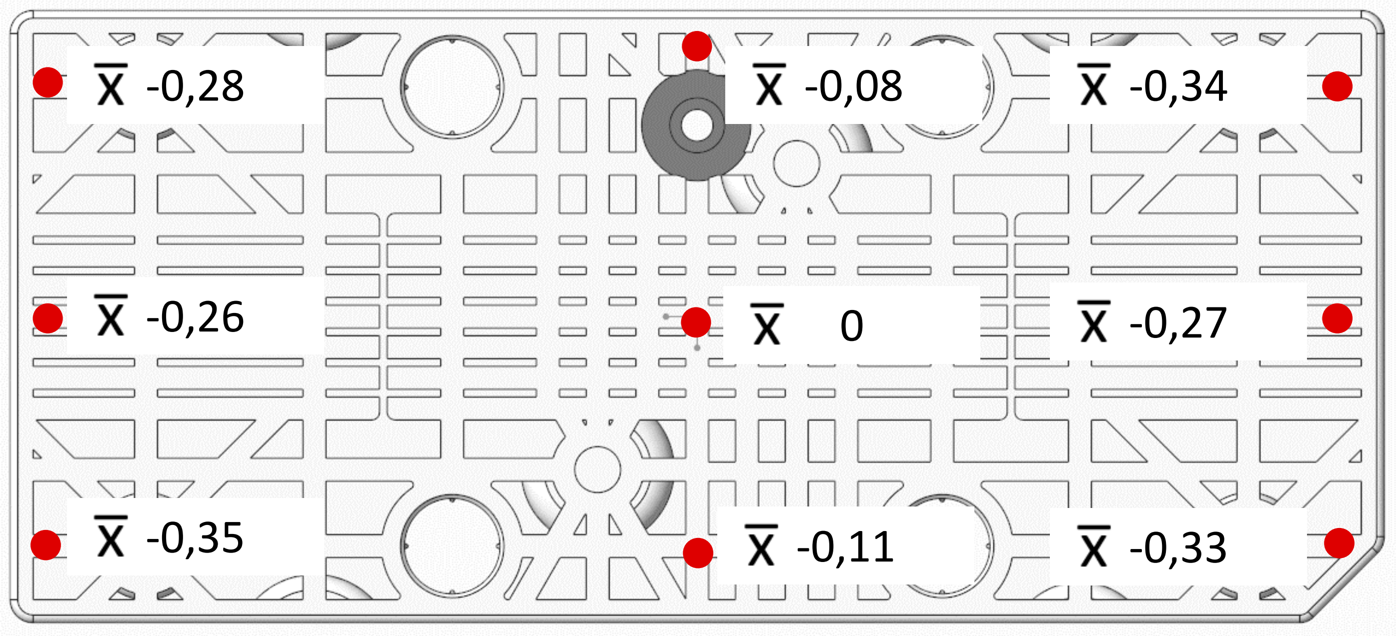


Determined evenness ≈ **0,35**

Tobias Vieten, Dennis Stahl, Peter Schilling, Faruk Civelek and André Zimmermann, Feasibility study of soft tooling inserts for injection molding with integrated automated slides, micromachines, 2021
